# (3a*RS*,7a*SR*)-7a-Meth­oxy-2-oxo-2,3,3a,4,5,6,7,7a-octa­hydro-1-benzofuran-4,4-dicarbonitrile

**DOI:** 10.1107/S1600536812048519

**Published:** 2012-11-30

**Authors:** María González, Andrea Martínez, Marcos L. Rivadulla, Berta Covelo

**Affiliations:** a Dpto. Química Orgánica, Facultade de Química, Universidade de Vigo, E-36310 Vigo, Spain; b Unidad de Difracción de Raios X de Monocristal, Servicio Determinación Estructural, Proteómica e Xenómica, CACTI-Universidade de Vigo, E-36310 Vigo, Spain

## Abstract

The racemic title compound, C_11_H_12_N_2_O_3_, contains a [4.3.0]bicyclic unit in which the shared C—C bond adopts a *cis* configuration. The five- and six-membered rings are in twisted envelope (with the bridgehead C atom bearing the methoxy substituent as the flap) and distorted chair conformations, respectively. In the crystal, the mol­ecules are linked *via* weak C—H⋯O iteractions, forming ladder-like chains along [010].

## Related literature

For related syntheses of natural products, see: Jones & Goodbrand (1977 ▶). For details of a synthesis using different starting materials, see: Alonso *et al.* (2005 ▶); Pérez *et al.* (2004 ▶, 2005 ▶). For a related structure, see: Grudniewska *et al.* (2011 ▶). For puckering parameters, see, Cremer & Pople (1975 ▶).
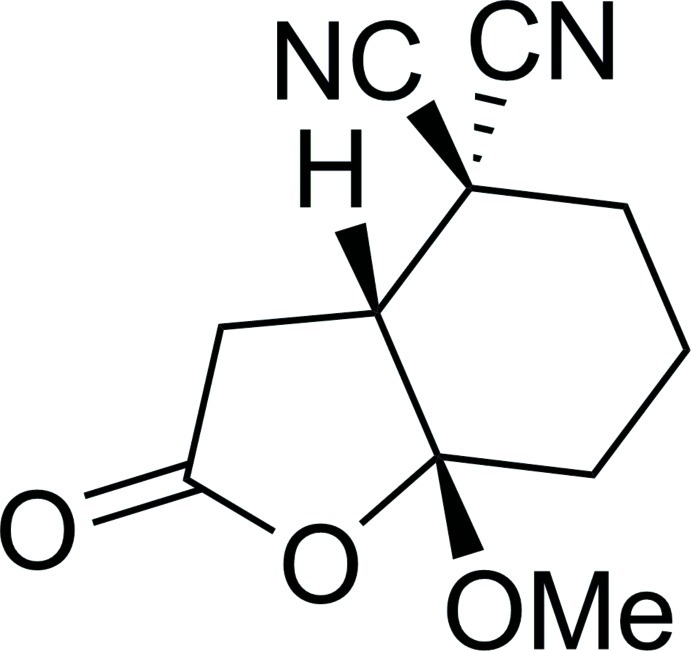


## Experimental

### 

#### Crystal data

C_11_H_12_N_2_O_3_
*M* *_r_* = 220.23
Monoclinic, 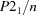

*a* = 11.816 (3) Å
*b* = 7.228 (2) Å
*c* = 13.017 (4) Å
β = 104.250 (5)°
*V* = 1077.6 (5) Å^3^
*Z* = 4
Mo *K*α radiation
μ = 0.10 mm^−1^
*T* = 293 K
0.49 × 0.24 × 0.19 mm

#### Data collection

Bruker SMART 1000 CCD diffractometer
Absorption correction: multi-scan (*SADABS*; Sheldrick, 1996 ▶) *T*_min_ = 0.697, *T*_max_ = 0.745
5473 measured reflections
1893 independent reflections
1244 reflections with *I* > 2σ(*I*)
*R*_int_ = 0.030

#### Refinement

*R*[*F* ^2^ > 2σ(*F* ^2^)] = 0.048
*wR*(*F* ^2^) = 0.143
*S* = 1.03
1893 reflections
146 parameters
H-atom parameters constrained
Δρ_max_ = 0.45 e Å^−3^
Δρ_min_ = −0.19 e Å^−3^

### 

Data collection: *SMART* (Bruker, 1998 ▶); cell refinement: *SAINT* (Bruker, 1998 ▶); data reduction: *SAINT*; program(s) used to solve structure: *SHELXS97* (Sheldrick, 2008 ▶); program(s) used to refine structure: *SHELXL97* (Sheldrick, 2008 ▶); molecular graphics: *PLATON* (Spek, 2011) ▶ and *Mercury* (Macrae *et al.*, 2006 ▶); software used to prepare material for publication: *SHELXTL* (Sheldrick, 2008 ▶).

## Supplementary Material

Crystal structure: contains datablock(s) I, global. DOI: 10.1107/S1600536812048519/jj2158sup1.cif

Structure factors: contains datablock(s) I. DOI: 10.1107/S1600536812048519/jj2158Isup2.hkl

Supplementary material file. DOI: 10.1107/S1600536812048519/jj2158Isup3.cml

Additional supplementary materials:  crystallographic information; 3D view; checkCIF report

## Figures and Tables

**Table 1:** Hydrogen-bond geometry (Å, °)

*D*—H⋯*A*	*D*—H	H⋯*A*	*D*⋯*A*	*D*—H⋯*A*
C3—H3*A*⋯O3^i^	0.97	2.59	3.290 (3)	129
C8—H8*C*⋯O2^ii^	0.96	2.46	3.219 (4)	136

Symmetry codes: (i) 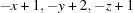; (ii) 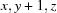.

**Fig. 1.**
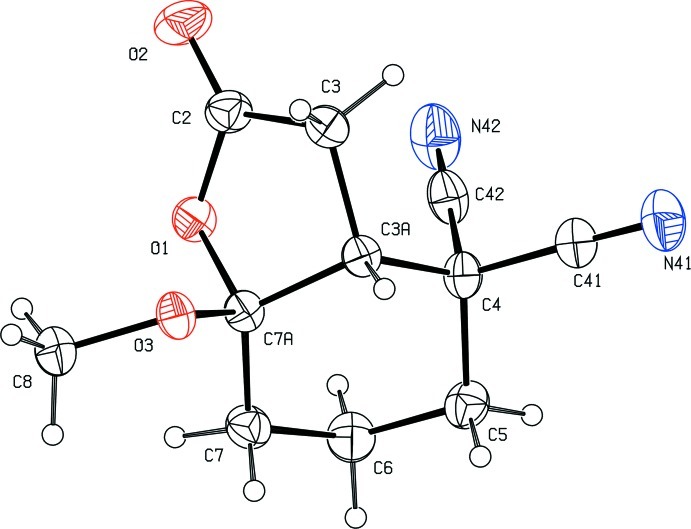
The molecular structure of the title compound. Non-H atoms are present as displacement ellipsoids at the 30% probability level.

**Fig. 2.**
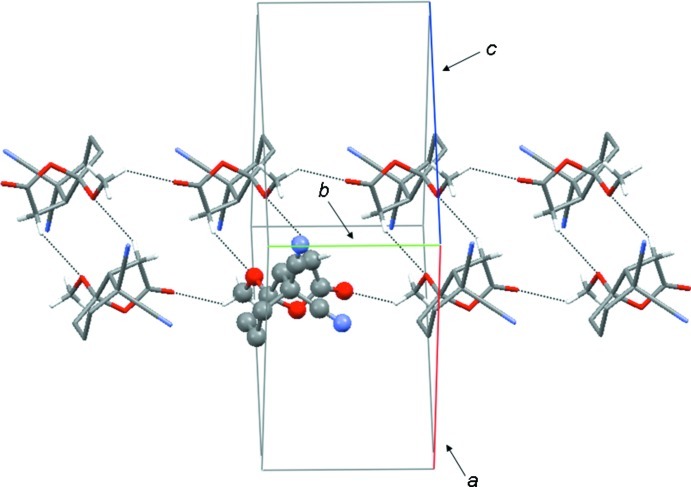
The crystal structure of the title compound. Weak C—H···O intermolecular interactions link the molecules into chains along [001]. H atoms not involved in hydrogen bonds (dashed lines) have been omitted for clarity.

## supplementary crystallographic information

### Comment

The racemic title compound is a [4.3.0] bicyclic γ-lactone obtained through an intramolecular Michael addition. The construction of carbocyclic systems is of paramount importance in organic synthesis since they are intermediate compounds for the preparation of interesting natural products (Jones *et al.*, 1977) as carbocyclic nucleosides and vitamin D analogues. We have described a new methodology for the synthesis of oxacyclic compounds using either methoxyallene (Alonso *et al.*, 2005) or furan (Pérez *et al.*, 2005) as a starting material. In the title compound (Fig. 1), the C—C share bond of the bicyclic moiety adopts a *cis* configuration. The 5-membered ring adopts a twisted envelope conformation with puckering parameters Q = 0.358 (2) Å and φ = 126.7 (4)° (Cremer & Pople, 1975) and the 6-membered ring adopts a distorted chair conformation with puckering parameters Q = 0.531 (3) Å, θ = 19.3 (3)° and φ = 123.3 (9)° (Cremer & Pople (1975). All bond lengths and bond angles are normal comparable to those observed in similar crystal structures (Grudniewska *et al.*, 2011). In the crystal structure, the molecules are self-assembled *via* weak C—H···O intermolecular interactions (Table 1) to form a ladderlike chain structure along [010] (Fig. 2).

### Experimental

To a solution of 2-(3-(2-methoxy-5-oxo-2,5-dihydrofuran-2-yl)propyl)malononitrile (0.38 mmol) in DMF (3 ml) was added DBU (0.19 mmol, 0.5 eq) and the mixture was stirred at room temperature. At the end of the reaction (TLC), EtOAc (20 ml) was added and the organic layers washed with water (3 *x* 20 ml), dried (Na_2_SO_4_), filtered, and concentrated to afford a residue, which was chromatographed on silica gel giving the title compound. It was then recrystallized using EtOAc/Hexane.

### Refinement

All H atoms were positioned geometrically and refined using a riding model with C—H = 0.96–0.98 Å and *U*_iso_(H) = 1.2*U*_eq_(C) or 1.5 *U*_eq_(C_methyl_).

### Crystal data

**Table:**

C_11_H_12_N_2_O_3_	*F*(000) = 464
*M**_r_* = 220.23	*D*_x_ = 1.357 Mg m^−^^3^
Monoclinic, *P*2_1_/*n*	Mo *K*α radiation, λ = 0.71073 Å
Hall symbol: -P 2yn	Cell parameters from 1285 reflections
*a* = 11.816 (3) Å	θ = 2.7–22.8°
*b* = 7.228 (2) Å	µ = 0.10 mm^−^^1^
*c* = 13.017 (4) Å	*T* = 293 K
β = 104.250 (5)°	Prism, colourless
*V* = 1077.6 (5) Å^3^	0.49 × 0.24 × 0.19 mm
*Z* = 4	

### Data collection

**Table:**

Bruker SMART 1000 CCD diffractometer	1893 independent reflections
Radiation source: fine-focus sealed tube	1244 reflections with *I* > 2σ(*I*)
Graphite monochromator	*R*_int_ = 0.030
φ and ω scans	θ_max_ = 25.0°, θ_min_ = 2.1°
Absorption correction: multi-scan (*SADABS*; Sheldrick, 1996)	*h* = −13→14
*T*_min_ = 0.697, *T*_max_ = 0.745	*k* = −8→8
5473 measured reflections	*l* = −14→15

### Refinement

**Table:**

Refinement on *F*^2^	Primary atom site location: structure-invariant direct methods
Least-squares matrix: full	Secondary atom site location: difference Fourier map
*R*[*F*^2^ > 2σ(*F*^2^)] = 0.048	Hydrogen site location: inferred from neighbouring sites
*wR*(*F*^2^) = 0.143	H-atom parameters constrained
*S* = 1.03	*w* = 1/[σ^2^(*F*_o_^2^) + (0.0722*P*)^2^ + 0.3369*P*] where *P* = (*F*_o_^2^ + 2*F*_c_^2^)/3
1893 reflections	(Δ/σ)_max_ < 0.001
146 parameters	Δρ_max_ = 0.45 e Å^−^^3^
0 restraints	Δρ_min_ = −0.19 e Å^−^^3^

### Special details

**Table:**

Experimental. [mp: 175–177 °C; IR (neat): 2248, 1800, 1744 cm^-1^; ^1^H NMR (CDCl_3_): δ: 3.38 (3*H*, s, OMe); 3.21 (1*H*, dd, J = 7.60, 17.78); 2.86 (1*H*, dd, J = 3.53, 7.60); 2.70 (1*H*, dd, J = 3.53, 17.78); 2.39–2.33 (2*H*, m); 2.16–1.80 (4*H*, m); ^13^C NMR (CDCl_3_): δ: 171.69 (C=O); 114.67 (CN); 112.89 (CN); 105.51 (C); 50.05 (OMe); 45.74 (CH); 35.79 (CH_2_); 33.59 (CH_2_); 31.89 (CH_2_); 28.56 (CH_2_); 17.68 (CH_2_); HRMS: calcd for C_11_H_13_N_2_O_3_ [*M*+1H]^+^ 221.0926, found 221.0583].
Geometry. All e.s.d.'s (except the e.s.d. in the dihedral angle between two l.s. planes) are estimated using the full covariance matrix. The cell e.s.d.'s are taken into account individually in the estimation of e.s.d.'s in distances, angles and torsion angles; correlations between e.s.d.'s in cell parameters are only used when they are defined by crystal symmetry. An approximate (isotropic) treatment of cell e.s.d.'s is used for estimating e.s.d.'s involving l.s. planes.
Refinement. Refinement of *F*^2^ against ALL reflections. The weighted *R*-factor *wR* and goodness of fit *S* are based on *F*^2^, conventional *R*-factors *R* are based on *F*, with *F* set to zero for negative *F*^2^. The threshold expression of *F*^2^ > σ(*F*^2^) is used only for calculating *R*-factors(gt) *etc*. and is not relevant to the choice of reflections for refinement. *R*-factors based on *F*^2^ are statistically about twice as large as those based on *F*, and *R*- factors based on ALL data will be even larger.

### Fractional atomic coordinates and isotropic or equivalent isotropic displacement parameters (Å^2^)

**Table:**

	*x*	*y*	*z*	*U*_iso_*/*U*_eq_	
O1	0.67913 (13)	0.7856 (2)	0.36232 (13)	0.0423 (5)	
O2	0.67361 (18)	0.5064 (3)	0.43402 (17)	0.0660 (6)	
O3	0.63413 (13)	1.0294 (2)	0.45900 (12)	0.0401 (4)	
N41	0.2043 (2)	0.7955 (4)	0.1943 (2)	0.0671 (7)	
N42	0.5146 (2)	0.5424 (4)	0.1555 (2)	0.0700 (8)	
C2	0.6249 (2)	0.6485 (4)	0.4044 (2)	0.0454 (6)	
C3	0.5029 (2)	0.7069 (3)	0.40461 (19)	0.0415 (6)	
H3A	0.4961	0.7318	0.4761	0.050*	
H3B	0.4467	0.6125	0.3731	0.050*	
C3A	0.48499 (18)	0.8826 (3)	0.33778 (17)	0.0344 (6)	
H3	0.4371	0.9708	0.3658	0.041*	
C4	0.42864 (19)	0.8445 (3)	0.21834 (18)	0.0394 (6)	
C5	0.4493 (2)	1.0080 (4)	0.14904 (19)	0.0464 (7)	
H5A	0.4119	0.9828	0.0753	0.056*	
H5B	0.4150	1.1195	0.1698	0.056*	
C6	0.5794 (2)	1.0369 (4)	0.1619 (2)	0.0508 (7)	
H6A	0.5916	1.1360	0.1155	0.061*	
H6B	0.6137	0.9250	0.1415	0.061*	
C7	0.6392 (2)	1.0854 (4)	0.27642 (19)	0.0453 (6)	
H7A	0.7230	1.0856	0.2845	0.054*	
H7B	0.6164	1.2097	0.2911	0.054*	
C7A	0.61086 (19)	0.9552 (3)	0.35683 (18)	0.0352 (6)	
C8	0.7522 (2)	1.0884 (4)	0.5017 (2)	0.0520 (7)	
H8A	0.8048	0.9939	0.4902	0.078*	
H8B	0.7645	1.1105	0.5764	0.078*	
H8C	0.7662	1.2004	0.4672	0.078*	
C42	0.4769 (2)	0.6739 (4)	0.1824 (2)	0.0483 (7)	
C41	0.3020 (2)	0.8146 (4)	0.2047 (2)	0.0460 (6)	

### Atomic displacement parameters (Å^2^)

**Table:**

	*U*^11^	*U*^22^	*U*^33^	*U*^12^	*U*^13^	*U*^23^
O1	0.0349 (9)	0.0400 (10)	0.0545 (10)	0.0055 (7)	0.0155 (8)	−0.0029 (8)
O2	0.0677 (13)	0.0411 (12)	0.0881 (16)	0.0172 (10)	0.0173 (11)	0.0123 (10)
O3	0.0359 (9)	0.0462 (10)	0.0375 (9)	−0.0036 (7)	0.0078 (7)	−0.0089 (7)
N41	0.0405 (14)	0.086 (2)	0.0718 (17)	−0.0063 (13)	0.0091 (12)	−0.0035 (14)
N42	0.0580 (16)	0.0740 (19)	0.0745 (18)	0.0034 (14)	0.0096 (13)	−0.0302 (15)
C2	0.0500 (15)	0.0367 (15)	0.0489 (15)	0.0027 (12)	0.0113 (12)	−0.0013 (12)
C3	0.0409 (14)	0.0411 (14)	0.0440 (14)	−0.0036 (11)	0.0133 (11)	−0.0006 (12)
C3A	0.0316 (12)	0.0379 (13)	0.0357 (13)	0.0007 (10)	0.0122 (10)	−0.0021 (10)
C4	0.0320 (12)	0.0472 (15)	0.0388 (13)	0.0008 (10)	0.0080 (10)	−0.0043 (11)
C5	0.0462 (15)	0.0574 (17)	0.0358 (14)	0.0016 (12)	0.0102 (11)	0.0048 (11)
C6	0.0494 (16)	0.0640 (19)	0.0424 (15)	−0.0038 (13)	0.0177 (12)	0.0042 (13)
C7	0.0438 (14)	0.0470 (15)	0.0468 (15)	−0.0060 (12)	0.0146 (11)	0.0027 (12)
C7A	0.0330 (12)	0.0357 (13)	0.0384 (13)	0.0012 (10)	0.0113 (10)	−0.0041 (10)
C8	0.0449 (15)	0.0523 (16)	0.0527 (16)	−0.0055 (13)	0.0003 (12)	−0.0030 (13)
C42	0.0392 (14)	0.0572 (18)	0.0473 (15)	−0.0049 (13)	0.0081 (12)	−0.0164 (14)
C41	0.0390 (15)	0.0535 (17)	0.0446 (15)	−0.0012 (12)	0.0086 (11)	−0.0042 (12)

### Geometric parameters (Å, º)

**Table:**

O1—C2	1.366 (3)	C4—C42	1.482 (4)
O1—C7A	1.460 (3)	C4—C5	1.542 (3)
O2—C2	1.194 (3)	C5—C6	1.519 (4)
O3—C7A	1.397 (3)	C5—H5A	0.9700
O3—C8	1.433 (3)	C5—H5B	0.9700
N41—C41	1.137 (3)	C6—C7	1.525 (4)
N42—C42	1.141 (3)	C6—H6A	0.9700
C2—C3	1.503 (3)	C6—H6B	0.9700
C3—C3A	1.524 (3)	C7—C7A	1.505 (3)
C3—H3A	0.9700	C7—H7A	0.9700
C3—H3B	0.9700	C7—H7B	0.9700
C3A—C7A	1.539 (3)	C8—H8A	0.9600
C3A—C4	1.558 (3)	C8—H8B	0.9600
C3A—H3	0.9800	C8—H8C	0.9600
C4—C41	1.479 (3)		
			
C2—O1—C7A	108.73 (17)	H5A—C5—H5B	108.2
C7A—O3—C8	115.36 (17)	C5—C6—C7	110.6 (2)
O2—C2—O1	121.2 (2)	C5—C6—H6A	109.5
O2—C2—C3	128.8 (2)	C7—C6—H6A	109.5
O1—C2—C3	110.0 (2)	C5—C6—H6B	109.5
C2—C3—C3A	103.38 (18)	C7—C6—H6B	109.5
C2—C3—H3A	111.1	H6A—C6—H6B	108.1
C3A—C3—H3A	111.1	C7A—C7—C6	114.0 (2)
C2—C3—H3B	111.1	C7A—C7—H7A	108.7
C3A—C3—H3B	111.1	C6—C7—H7A	108.7
H3A—C3—H3B	109.1	C7A—C7—H7B	108.7
C3—C3A—C7A	101.50 (17)	C6—C7—H7B	108.7
C3—C3A—C4	112.80 (19)	H7A—C7—H7B	107.6
C7A—C3A—C4	112.33 (17)	O3—C7A—O1	107.39 (18)
C3—C3A—H3	110.0	O3—C7A—C7	113.4 (2)
C7A—C3A—H3	110.0	O1—C7A—C7	110.14 (18)
C4—C3A—H3	110.0	O3—C7A—C3A	103.91 (17)
C41—C4—C42	107.3 (2)	O1—C7A—C3A	102.82 (18)
C41—C4—C5	110.1 (2)	C7—C7A—C3A	118.23 (19)
C42—C4—C5	108.9 (2)	O3—C8—H8A	109.5
C41—C4—C3A	108.48 (18)	O3—C8—H8B	109.5
C42—C4—C3A	111.02 (19)	H8A—C8—H8B	109.5
C5—C4—C3A	110.99 (19)	O3—C8—H8C	109.5
C6—C5—C4	110.0 (2)	H8A—C8—H8C	109.5
C6—C5—H5A	109.7	H8B—C8—H8C	109.5
C4—C5—H5A	109.7	N42—C42—C4	179.5 (3)
C6—C5—H5B	109.7	N41—C41—C4	178.6 (3)
C4—C5—H5B	109.7		
			
C7A—O1—C2—O2	−166.8 (2)	C8—O3—C7A—C3A	173.04 (19)
C7A—O1—C2—C3	13.8 (3)	C2—O1—C7A—O3	77.4 (2)
O2—C2—C3—C3A	−169.1 (3)	C2—O1—C7A—C7	−158.74 (19)
O1—C2—C3—C3A	10.3 (3)	C2—O1—C7A—C3A	−31.9 (2)
C2—C3—C3A—C7A	−28.3 (2)	C6—C7—C7A—O3	−160.9 (2)
C2—C3—C3A—C4	92.2 (2)	C6—C7—C7A—O1	78.7 (2)
C3—C3A—C4—C41	78.0 (2)	C6—C7—C7A—C3A	−39.0 (3)
C7A—C3A—C4—C41	−168.0 (2)	C3—C3A—C7A—O3	−75.5 (2)
C3—C3A—C4—C42	−39.6 (2)	C4—C3A—C7A—O3	163.78 (18)
C7A—C3A—C4—C42	74.3 (2)	C3—C3A—C7A—O1	36.4 (2)
C3—C3A—C4—C5	−160.97 (18)	C4—C3A—C7A—O1	−84.4 (2)
C7A—C3A—C4—C5	−47.0 (3)	C3—C3A—C7A—C7	157.9 (2)
C41—C4—C5—C6	−179.2 (2)	C4—C3A—C7A—C7	37.2 (3)
C42—C4—C5—C6	−61.8 (3)	C41—C4—C42—N42	−121 (39)
C3A—C4—C5—C6	60.7 (3)	C5—C4—C42—N42	120 (39)
C4—C5—C6—C7	−61.9 (3)	C3A—C4—C42—N42	−3 (40)
C5—C6—C7—C7A	50.4 (3)	C42—C4—C41—N41	−160 (11)
C8—O3—C7A—O1	64.6 (2)	C5—C4—C41—N41	−42 (11)
C8—O3—C7A—C7	−57.3 (3)	C3A—C4—C41—N41	80 (11)

### Hydrogen-bond geometry (Å, º)

**Table:**

*D*—H···*A*	*D*—H	H···*A*	*D*···*A*	*D*—H···*A*
C3—H3*A*···O3^i^	0.97	2.59	3.290 (3)	129
C8—H8*C*···O2^ii^	0.96	2.46	3.219 (4)	136

Symmetry codes: (i) −*x*+1, −*y*+2, −*z*+1; (ii) *x*, *y*+1, *z*.
